# Abnormal TNS3 gene methylation in patients with congenital scoliosis

**DOI:** 10.1186/s12891-022-05730-x

**Published:** 2022-08-20

**Authors:** YuanTao Wu, Hong-qi Zhang, Mingxing Tang, Chaofeng Guo, Shaohua Liu, Jiong Li, Yunjia Wang, Lige Xiao, Guanteng Yang

**Affiliations:** 1 Department of Spine Surgery, Hainan General Hospital and Hainan Affiliated Hospital of Hainan Medical University, Haikou, China; 2 Department of Spine Surgery, Xiangya Hospital of Central-South University, Hunan, 410008 China

**Keywords:** Congenital scoliosis, Genome methylation sequencing, Methylation level of the promoter region, MethylTarget, TNS3, Immunohistochemistry

## Abstract

**Background:**

Congenital scoliosis (CS) is a congenital deformity of the spine resulting from abnormal and asymmetrical development of vertebral bodies during pregnancy. However, the etiology and mechanism of CS remain unclear. Epigenetics is the study of heritable variations in gene expression outside of changes in nucleotide sequence. Among these, DNA methylation was described first and is the most characteristic and most stable epigenetic mechanism. Therefore, in this study, we aim to explore the association between genome methylation and CS which are not been studied before.

**Methods:**

Two pairs of monozygotic twins were included, with each pair involving one individual with and one without CS. Agilent SureSelect XT Human Methyl-Sequencing was used for genome methylation sequencing. MethylTarget was used to detect methylation levels in target regions. Immunohistochemistry was performed to visualize expression of associated genes in candidate regions.

**Results:**

A total of 75 differentially methylated regions were identified, including 24 with an increased methylation level and 51 with a decreased methylation level in the CS group. Nine of the differentially methylated regions were selected (*TNS3*, *SEMAC3*, *GPR124*, *MEST*, *DLK1*, *SNTG1*, *PPIB*, *DEF8*, and *GRHL2*). The results showed that the methylation level of the promoter region of *TNS3* was 0.72 ± 0.08 in the CS group and 0.43 ± 0.06 in the control group (*p* = 0.00070 < 0.01). There was no significant difference in the degree of methylation of *SEMAC3*, *GPR124*, *MEST*, *DLK1*, *SNTG1*, *PPIB*, *DEF8*, or *GRHL2* between the two groups. Immunohistochemistry showed significantly decreased TNS3 expression in the cartilage of the articular process in CS (CS: 0.011 ± 0.002; control: 0.018 ± 0.006, *P* = 0.003 < 0.01).

**Conclusion:**

Compared with the control group, high-level methylation of the *TNS3* promoter region and low TNS3 expression in the cartilage layer of the articular process characterize CS. Thus, DNA methylation and TNS3 may play important roles in the pathogenesis of CS.

**Supplementary Information:**

The online version contains supplementary material available at 10.1186/s12891-022-05730-x.

## Background

Congenital scoliosis (CS) is a congenital deformity of the spine resulting from abnormal and asymmetrical development of vertebral bodies during pregnancy (usually between 4 and 6 gestational weeks). The common manifestation is deformity of a single or multiple vertebral bodies, which can be combined with rib fusion, malposition, absence, diastematomyelia, tethered spinal cord, and malformations of other systems; the incidence fluctuates between 0.5‰ and 1‰, accounting for approximately 5.19% of all spinal deformities [1–3]. In clinical practice, different types of CS share characteristics of early onset, rapid progression, severe deformity, and many complications, which seriously affect the life of patients and may even lead to cardiopulmonary dysfunction and neurological impairment when the deformity is severe. There are few treatment methods, though the efficacy of brace treatment is poor, rendering surgery the only solution for severe CS [4]. Due to the large number of patients and limited number of treatment methods, this disease seriously affects the physical and mental health of patients [5, 6]. Therefore, it is of great significance to actively explore the etiology and pathogenesis of CS and to fundamentally curb the occurrence and development of the disease.

However, the etiology and mechanism of CS are still unclear. Most scholars suggest that disease onset is mainly due to the joint action of genetic factors and environmental factors [7–9]. Epigenetics is the study of heritable variations in gene expression outside of changes in nucleotide sequence. Among these, DNA methylation has been known for the longest time, is the most characteristic, and is most stable epigenetic mechanism [10]. Many complex diseases, such as adolescent idiopathic scoliosis (AIS), are associated with DNA methylation. For example, Mao et al. found that in AIS, the promoter region of *COMP* shows a high level of methylation, leading to low expression of the gene, and a high level of methylation correlated with the size of the Cobb angle and age of onset [11]. A high level of methylation in the promoter of *PITX1* has also been detected AIS patients [12]. Meng et al. found that DNA methylation is not only associated with AIS pathogenesis but can also be used as a predictor of curve progression [13]. Thus, DNA methylation may be involved in complex diseases.

Although methylation is well studied in various complex diseases, its association with CS has not been examined. In this article, we explore promoter region methylation in CS and clarify whether DNA methylation plays a role in the pathogenesis of CS to possibly provide a new explanation for its pathogenesis.

## Methods

### Ethical statement

This study was approved by the Ethics Committee of Xianya Hospital (No. 201703358) and complied with the 1964 Helsinki Declaration and its later amendments or comparable ethical standards. Informed consent was obtained from all patients and controls and their legal guardians.

### Subjects and samples

All patients had received treatment at our hospital between September 2016 and September 2018. In the first phase, namely, the discovery stage, two pairs of monozygotic twins were identified. In the second phase, the methylation of the target region stage, 50 CS and 50 control were enrolled. In the third phase, the gene expression stage, 20 CS and 20 control specimens were collected for validation. The inclusion criteria for the CS and control groups were as follows: children who signed informed consent; complete clinical data and imaging data; first diagnosed with CS in our hospital based on medical history, physical examination, and auxiliary examination without any prior treatment; and belonging to the Han Chinese population. Immunohistochemistry samples were surgically obtained, and children younger than 10 years who underwent spinal surgery but were not patients with spinal deformities were rare; thus, patients aged 10 to 18 years with lumbar disc herniation, thoracolumbar fractures, and spondylolisthesis were selected as controls. Patients with CS who were 10 to 18 years old were selected as the case group for the immunohistochemical analysis. The exclusion criteria for both groups were lack of informed consent, a clear history of the mother taking medication or toxic substances during the pregnancy, incomplete data for the patient, history of treatment with a spinal brace, and presence of any of various syndromes. Detailed information is listed in Table 1.

**Table 1:** Clinical data

Subject	Number (male/female)	Age(y)	Height(m)	Weight(kg)	Type			Position			Cobb(°)
					I	II	III	cervical	thoracic	lumbar	
**The First Stage**											
Pair 1											
C26	Male	15	1.63	45			1		1		88.9
N12	Male	15	1.78	63							
Pair 2											
C2	Male	4	0.96	14	1				1		50.2
N1	Male	4	1.09	19							
**The Second Stage**											
Case	50 (31/19)	10.3 ± 3.7	1.2 ± 0.1	23.2 ± 6.5	35	8	7	2	30	18	40.5 ± 13.9
Control	50 (30/20)	11.3 ± 3.3	1.4 ± 0.1	33.6 ± 7.2							
* p* value^*^	0.84	0.157	** < 0.01**	** < 0.01**							
**The Third Stage**											
Case	20(8/12)	14.5 ± 1.8	1.51 ± 0.1	40.22 ± 5.15	11	4	5		11	9	50.5 ± 18.9
Control	20(13/7)	15.1 ± 1.3	1.59 ± 0.13	46.33 ± 7.4							
* P* value	0.113	0.234	**0.035**	** < 0.01**							

Values in Bold indicates *p* value < 0.05, which is considered as significance difference

^*^By independent sample t test

### Agilent sureselect xt human methyl-sequencing [14]

DNA was extracted from peripheral blood using SQ Blood DNA Kit II (OMEGA Bio-TEK, USA). For target bisulfite sequencing library construction, we used NimbleGen SeqCap Epi Enrichment System to capture target genomic regions. The experiment involves several steps, as follows. [1] Genomic DNA: The NimbleGen SeqCap Epi oligo pool was prepared against target regions in the genome. [2] Library Preparation: A standard shotgun sequencing library with methylated adapters was generated from genomic DNA. [3] Bisulfite Conversion: The sequencing library was treated with bisulfite to convert unmethylated cytosines to uracil. [4] Hybridization: The bisulfite-treated sequencing library was hybridized to the SeqCap Epi oligo pool. [5] Bead capture: Capture beads were used to pull down the complex of capture oligos and genomic DNA fragments. [6] Washing: Unbound fragments were removed by washing. [7] Amplification: The enriched fragment pool was amplified by PCR. [8] Bisulfite sequencing-ready DNA: The enriched fragment pool was amplified by PCR.

Putative different methylation regions in gene promoters were identified by comparison of the case and control methylomes using windows that contained at least 5 CpG (CHG or CHH) sites with a twofold change in methylation level. Two nearby differentially methylated regions (DMR) would be considered interdependent and joined into one continuous DMR if the genomic region from the start of an upstream DMR to the end of a downstream DMR also showed twofold methylation level differences between the case and control groups. Otherwise, the two DMRs were viewed as independent. After iteratively merging interdependent DMRs, the final dataset of tDMRs comprised those that were independent from each other.

### Methylation of the target region

The target region was selected to design primers. First, net-PCR was used to amplify the DNA of the target region, after which an Illumina HiSeq 200 was used to sequence the DNA fragments. After methylation calling, data with a bisulfite conversion rate < 98% were excluded; data with an average CpG island coverage less than 20 × and a loss rate greater than 0.20 were removed after preliminary analysis. Finally, samples with a missed detection rate greater than 0.30 were removed.

### Immunohistochemistry

Articular processes were obtained from the CS and control groups, fixed in formalin, decalcified, and embedded in paraffin. After sectioning, rehydration was performed. The sections were rinsed in xylene twice for 15 min each, in gradient ethanol solutions once (100%, 95%, 85%, and 70% for 5 min each), and in double-distilled water (ddH_2_O) three times for 3 min each and then treated with 3% hydrogen peroxide for 5 min. The sections were incubated with 3% bovine serum albumin for 30 min at room temperature and then incubated with the primary antibody (TNS3) at 37 °C overnight. The sections were then incubated with a biotinylated goat anti-rabbit antibody for 30 min. Afterwards, the sections were incubated for 50 min with horseradish peroxidase-labeled streptavidin. DAB chromogen was added for 30 s for color development. Finally, the sections were counterstained with hematoxylin for 30 s, and the slide was mounted for observation.

### Statistics

Results were recorded and analyzed by SPSS software (version 24.0; SPSS, Inc., Chicago, IL, USA). Clinical and biological data were assessed by independent t tests and chi-square tests. Quantitative data were assessed by independent t test, and the results are expressed as the mean ± standard deviation. Numeration data were assessed by the chi-square test. In the first stage, the difference in DNA methylation between the case and control groups was compared using Fisher’s test. In the second stage, the difference in DNA methylation between the case and control groups was compared using the U test. A difference was considered significant if the *p* value was < 0.05.

## Results

The study was divided into three stages: in the first stage, location of regions of differential methylation were identified; in the second stage, sites of differential methylation were verified; and in the third stage, target gene expression was assessed.

Twin inclusion and screening for regions of differential methylation.

**Fig. 1:**
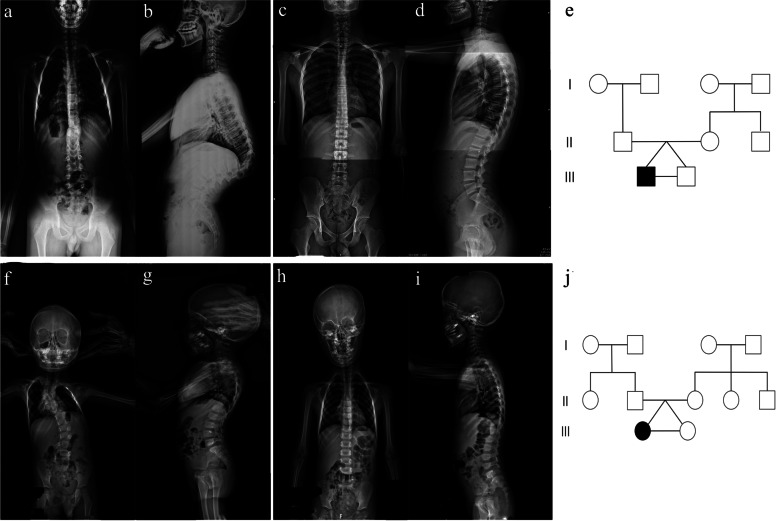
Imaging data and pedigree chart of twins in the first stage**. a, b** Anteroposterior and lateral positions of the CS patient of the first pair of twins, showing T10 hemivertebra, T11-12 local fusion, and a kyphotic angle of 88.9°. **c, d** Anteroposterior and lateral positions of the control in the first pair of twins, showing normal spinal morphology. **e** Pedigree chart of the first pair of twins. **f, g ** Anteroposterior and lateral positions of the CS patient of the second pair of twins, showing T10 hemivertebra, C3-6/T3 butterfly vertebra, T4-5 local fusion, and a Cobb angle of 39.7°. **h, i** Anteroposterior and lateral positions of the control in the second pair of twins, showing normal spinal morphology. **j** Pedigree chart of the second pair of twins

**Fig. 2:**
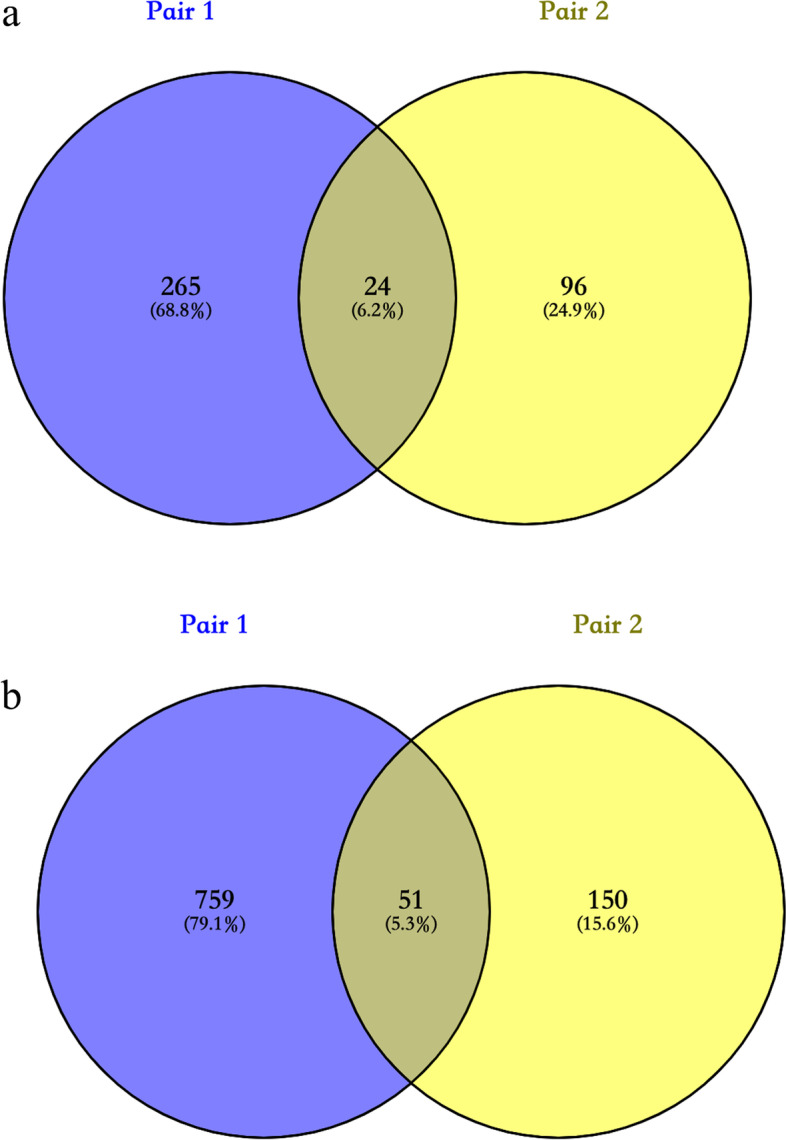
Genome methylation sequencing results for the twins in the first stage**. a** Venn diagram showing 24 regions in the CS group more highly methylated (> twofold) than the corresponding regions in the control group. **b** Venn diagram showing 51 regions in the CS group less methylated (< 0.5-fold) than the corresponding regions in the control group

Two pairs of monozygotic twins were included. Pair 1 included 15-year-old boys: a patient referred to as C1 and an unaffected brother referred to as N1. Pair 2 involved 4-year-old girls: a patient referred to as C2 and an unaffected sister referred to as N2 (Fig. 1). When comparing the difference in regional methylation levels between the CS group and the control group, the screening conditions were that a region in the CS group was considered differentially methylated if the degree of methylation was more than twice or less than half that of the corresponding region in the control group and if the *p* value for the comparison was less than 0.05. A total of 75 differentially methylated regions were identified: 24 with a higher methylation level and 51 with a lower methylation level in the CS group (Fig. 2). Considering that CS is mainly related to somite development, we reviewed the functions of genes in those regions and focused on those associated with osteogenic development, neural development, and ciliary development. We selected 9 differentially methylated regions as candidate sites (Table 2) and designed primers (Table 3) for validation in the second stage.

**Table 2:** Differentially methylated regions

Position	Differential Methylation Region Start	Differential Methylation Region End	*P* Value	Nromal Methylation	CS Methylation	Gene ID	Symbol	Log2 Ratio(C/N)
chr7	47,622,153	47,622,200	0.011	0.5617	0.79	64,759	TNS3	0.492
chr7	80,549,131	80,549,364	0.00084	0.60842	0.36625	10,512	SEMA3C	-0.732
chr8	37,653,890	37,653,973	0.0068	0.7515	0.8664	25,960	GPR124	0.205
chr7	130,130,288	130,130,549	0.0029	0.66906	0.55711	4232	MEST	-0.487
chr14	101,192,682	101,192,751	0.00073	0.7755	0.8962	8788	DLK1	0.209
chr8	50,824,309	50,824,417	0.0004	0.069437	0.1755	54,212	SNTG1	1.338
chr15	64,456,244	64,456,426	0.013	0.51943	0.37429	5479	PPIB	-0.473
chr16	90,014,349	90,014,430	0.0089	0.1555	0.3232	54,849	DEF8	1.056
chr8	102,504,447	102,504,502	0.023	0.1583	0.0404	79,977	GRHL2	-1.97

**Table 3:** Designed primers

Primer name	Primer
TNS3-F	ACACACAGCAGCCCTCTCC
TNS3-R	GGAGAAGGAGTGGACGGTGAG
SEMA3C-F	ACCACCAAGGAGTTCCCAGATG
SEMA3C-R	AGGCAGCAGTCAGCACAGG
GPR124-F	GTCATCTTCGCAGGAACCAGTG
GPR124-R	TAGTCCACACCAGCCTTTCCC
MEST-F	CTGTGGGTGTGGTTGGAAGTC
MEST-R	AGGCAGAGCAGCAGCAAGG
DLK1-F	GCAAGCCCGAGTTCACAGG
DLK1-R	GCGGCGGCAGATTCATTGG
SNTG1-F	CTCTCTGCTGAAGACTGCGTTG
SNTG1-R	CGGCTCCTTGCTTCCTCTGG
PPIB-F	TCCGTCTTCTTCCTGCTGCTG
PPIB-R	GGTGTCTTTGCCTGCGTTGG
DEF8-F	AGCAGTCGGAGAAGCAGAAGG
DEF8-R	CACTCGGCACAGCGGTAATC
GRHL2-F	GAGTGTGGTGATGGTGGTCTTC
GRHL2-R	CCCTTTCTTCCTGTTCTGCTTCC

### Validation of differentially methylated regions

**Fig. 3:**
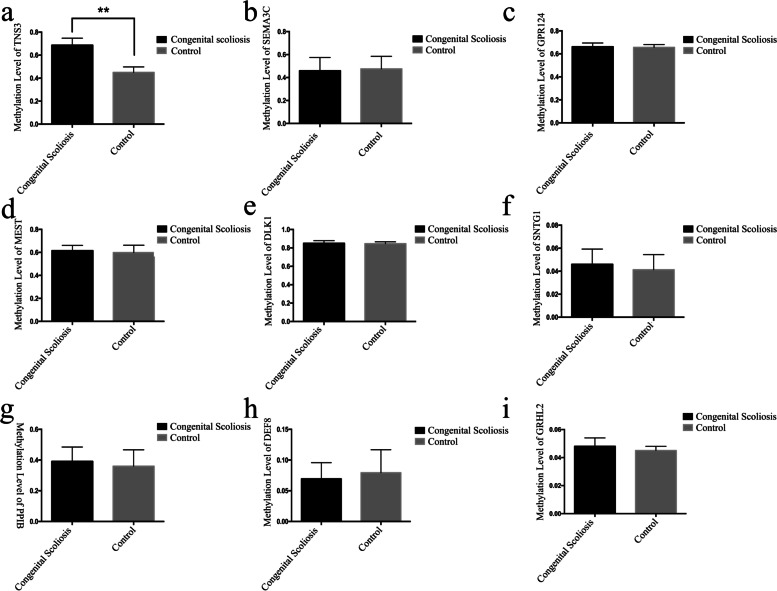
Methylation validation results of target regions for expanded validation in the second stage (50 CS vs 50 Control)**. a** The degree of methylation in the *TNS3* promoter region was significantly increased in CS patients versus the control group. **b, c, d, e, f, g, h, i** The degrees of methylation in the promoter regions of *SEMAC3*, *GPR124*, *MEST*, *DLK1*, *SNTG1*, *PPIB*, *DEF8*, and *GRHL2* in the CS group and the control group, for which between-group differences were not statistically significant. A *p* value < 0.05 denotes a significant difference between the two groups (**)

In the first stage, a total of 9 differentially methylated regions were selected as candidate regions for expanded validation. The results showed that the methylation level of the promoter region of *TNS3* was 0.72 ± 0.08 in the CS group and 0.43 ± 0.06 in the control group (*p* = 0.00070 < 0.01). No significant difference in the degree of methylation was found at *SEMA3C* (*p* = 0.41), *GPR124* (*p* = 0.43), *MEST* (*p* = 0.26), *DLK1* (*p* = 0.10), *SNTG1* (*p* = 0.080), *PPIB* (*p* = 0.13), *DEF8* (*p* = 0.33), or *GRHL2* (*p* = 0.074) between the two groups (Fig. 3).

### Immunohistochemistry

**Fig. 4:**
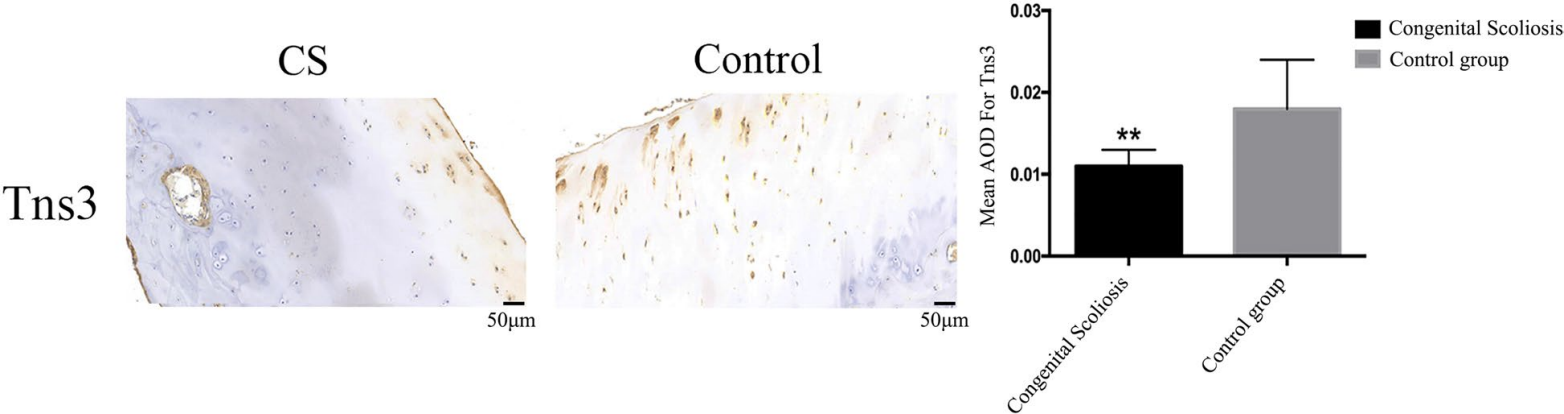
Immunohistochemical staining for TNS3 (20 CS vs 20 Control)**.** Representative image of immunohistochemical staining for TNS3 in the congenital scoliosis and control groups. Quantitative analysis of the mean intensity for positively stained areas. Data are shown as the mean ± SD. An independent t test was used; a *p* value < 0.05 denotes a significant difference between the two groups (**)

To further characterize expression of the above genes in CS, we included 20 patients with sporadic CS as the CS group and 20 age- and sex-matched surgical patients with no spinal malformities as the control group. TNS3 mainly promotes the proliferation of cartilage, whereas the growth of the articular process mainly depends on the osteogenic mode of endochondral ossification. Therefore, our main observation area was the cartilage area of the articular process. Expression of TNS3 in the CS group was significantly lower than that in the control group (Fig. 4) (CS: 0.011 ± 0.002; control: 0.018 ± 0.006, *p* = 0.003 < 0.01).

## Discussion

The earliest concept of epigenetics was proposed by Conrad Waddington in 1940. It emphasizes interactions between environmental factors and genetic factors, which jointly influence the phenotype of an organism. Epigenetics plays roles in various complex diseases, including AIS. Especially in recent years, studies on AIS suggest a possible link between DNA methylation and spinal deformity. Researchers have found that the promoter regions of bone development-related *COMP* and hormone secretion-related *PITX1* show high levels of methylation in AIS; such high levels of methylation would reduce their expression levels [11, 12]. Meng et al. performed methylation sequencing by including two pairs of twins, further expanded the inclusion of progressive and nonprogressive forms of AIS, and evaluated the predictive role of the cg01374129 methylation site in AIS progression, the sensitivity and specificity of which for predicting AIS progression reached 69% and 84%, respectively [13]. All these findings indicate that DNA methylation may play a role in the pathogenesis and progression of spinal deformities.

Considering that many scholars are currently studying homologs of disease genes of AIS and CS and CS is mainly caused by the joint actions of genetic factors and environmental factors, we enrolled two pairs of monozygotic twins in clinical practice, performed Agilent SureSelect XT Human Methyl-Sequencing, and carried out expanded validation experiments. To the best of our knowledge, this is the first study to report differential methylation in CS. In the first stage, a total of 75 promoter regions in the CS group showed methylation levels more than twice or less than half (*p* < 0.05) of the levels of the corresponding promoter regions in the control group: 24 with a higher and 51 with a lower methylation level. In the validation stage, the degree of *TNS3* methylation showed a statistically significant difference (1.67-fold) between groups in the expanded sample.

Tensins are a focal adhesion family with four members: TNS1, TNS2, TNS3, and C-terminal tensin-like (CTEN). TNS3 is widely expressed in various tissues of the body, including the thyroid, kidney, lungs, pancreas, and spleen, and may be involved in actin remodeling, cell migration, and skeletal development. Similar to TNS1, TNS3 contains Src homology 2, actin-binding, and phosphotyrosine-binding regions [15, 16]. TNS3 is a negative regulator of cell migration that can affect the cytoskeleton by modulating membrane receptor signaling pathways [17–19]. In a recent animal study, mice with *TNS3* knockout showed growth retardation, weight loss, and elevated mortality, which is similar to mice with CS [20]. In another study, researchers found that TNS3 expression was significantly increased during osteoclast differentiation and that inhibition of TNS3 resulted in decreased osteoclast activity as well as blocked bone resorption, clarifying the important role of TNS3 in bone metabolism [21]. These results suggest that TNS3 plays an important role in growth, development and bone metabolism. Our results showed that the degree of methylation in the promoter region of *TNS3* was significantly increased in CS; expression of TNS3 in the chondrocytes of the articular process in CS patients was also significantly reduced. These results indicate that although TNS3 may not be involved in the three important signaling pathways affecting somite formation, it may play an important role in regulating cell adhesion in late pregnancy or the early postnatal period, thereby maintaining normal skeletal growth, bone metabolism, and the development of other organs. In particular, reduced expression of TNS3 will maintain tension-deprived chondrocytes in a quiescent state during the development of the skeleton, resulting in slowed endochondral ossification and fewer chondrocytes in the proliferative zone; this in turn leads to abnormal development of the vertebral bodies. This is similar to the conclusions about TNS3 found for Drosophila and mice: TNS3 maintains the vital function of organ development by increasing cell adhesion capacity at specific developmental stages.

There are several limitations to this study. First, the sample size for the discovery stage was small, in part because MZ twins for CS are rare. Second, we only identified aberrant methylation in the promoter region of *TNS3* and aberrant protein expression of TNS3 in CS, with expanded sample validation and immunohistochemistry. Further studies should investigate how this gene is involved in the occurrence and development of CS.

## Conclusion

Compared with the control group, CS patients show a high level of methylation in the *TNS3* promoter region and low TNS3 expression in the cartilage layer of the articular process. These phenomena suggest that *TNS3* gene methylation and the TNS3 protein play an important role in the pathogenesis of CS.

## Supplementary Information

**Additional file 1.**

## Data Availability

All data generated or analysed during this study are included in this article.
