# Supplementary figures and images for: Abnormal TNS3 gene methylation in patients with congenital scoliosis
Source: BMC Musculoskelet Disord. 2022 Aug 20;23:797. doi: 10.1186/s12891-022-05730-x (PMC9392296; doi:10.1186/s12891-022-05730-x)

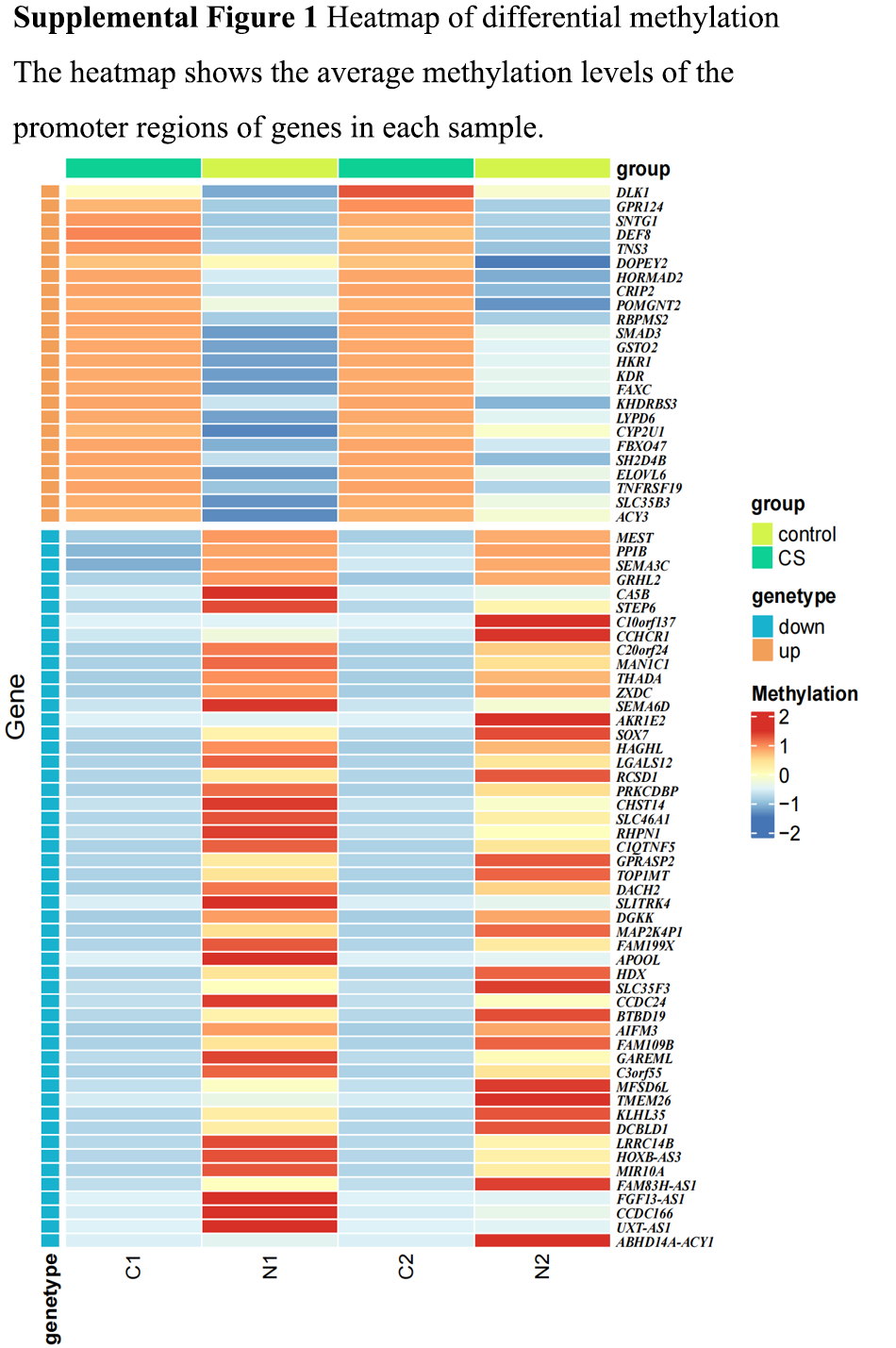

Supplement: Supplementary file 1 — Additional file 1. [file 12891_2022_5730_MOESM1_ESM.tif]
